# Identity Resolution in the Context of Dementia Caregiving: A Longitudinal Study

**DOI:** 10.1093/geroni/igaf122.2978

**Published:** 2025-12-31

**Authors:** Lauren Mitchell, Miriam Likhterman, Laura Sanchez Concepcion, Shaye Child, Zena Halwani, Grace Kavanagh

**Affiliations:** Emmanuel College, Boston, Massachusetts, United States; Emmanuel College, Boston, Massachusetts, United States; Emmanuel College, Boston, Massachusetts, United States; Emmanuel College, Boston, Massachusetts, United States; Emmanuel College, Boston, Massachusetts, United States; Emmanuel College, Boston, Massachusetts, United States

## Abstract

Becoming a caregiver for a loved one with dementia involves identity adjustment as caregivers’ self-concept shifts to accommodate their new role. Most research on caregivers’ identities has relied on qualitative methods; there is a need for quantitative, longitudinal research on caregivers’ identities over time. The present study used a validated measure rooted in Erikson’s (1968) classic theory of identity development to estimate trajectories for caregivers’ identity resolution. Caregivers (N = 81) of individuals with mild-to-moderate cognitive impairment completed the identity subscale of the Erikson Psychosocial Stage Inventory (Rosenthal et al., 1981), assessing overall sense of self-continuity and identity coherence, at 0, 3, and 6 months. Latent growth curve models estimated trajectories of change in identity resolution. Measures of caregiving tenure, depressive symptoms, and caregiver burden were included as moderators. Results indicated that caregivers on average experienced a high, stable level of identity resolution (I = 3.95, VarI=.34, S=-.001, VarS=.004). Time spent as a caregiver was not associated with level or change in identity resolution. Caregivers with greater depressive symptoms and higher burden at baseline experienced consistently lower identity resolution across the six-month period. Overall, caregivers’ identities seem to be highly resilient. Despite the identity-related challenges documented in qualitative literature (e.g., new responsibilities; changing relationship to the care recipient), caregivers may dynamically adapt and retain a high level of self-continuity and a clear, coherent self-concept. Conversely, caregivers who experience depression and high burden may benefit from support focused directly on identity adjustment.

